# Heat Stress Reduces Sperm Motility via Activation of Glycogen Synthase Kinase-3α and Inhibition of Mitochondrial Protein Import

**DOI:** 10.3389/fphys.2017.00718

**Published:** 2017-09-22

**Authors:** Yabin Gong, Huiduo Guo, Zhilong Zhang, Hao Zhou, Ruqian Zhao, Bin He

**Affiliations:** ^1^Key Laboratory of Animal Physiology and Biochemistry, Ministry of Agriculture, Nanjing Agricultural University Nanjing, China; ^2^Jiangsu Collaborative Innovation Center of Meat Production and Processing, Quality and Safety Control Nanjing, China

**Keywords:** heat stress, glycogen synthase kinase-3α, oxidative phosphorylation, mitochondrial remodeling, sperm

## Abstract

The adverse effects of high environmental temperature exposure on animal reproductive functions have been concerned for many decades. However, the molecular basis of heat stress (HS)-induced decrease of sperm motility has not been entirely elucidated. We hypothesized that the deteriorate effects of HS may be mediated by damage of mitochondrial function and ATP synthesis. To test this hypothesis, we use mature boar sperm as model to explore the impacts of HS on mitochondrial function and sperm motility. A 6 h exposure to 42°C (HS) induced significant decrease in sperm progressive motility. Concurrently, HS induced mitochondrial dysfunction that is indicated by decreased of membrane potential, respiratory chain complex I and IV activities and adenosine triphosphate (ATP) contents. Exogenous ATP abolished this effect suggesting that reduced of ATP synthesis is the committed step in HS-induced reduction of sperm motility. At the molecular level, the mitochondrial protein contents were significantly decreased in HS sperm. Notably, the cytochrome c oxidase subunit 4, which was synthesized in cytoplasm and translocated into mitochondria, was significantly lower in mitochondria of HS sperm. Glycogen synthase kinase-3α (GSK3α), a negative regulator of sperm motility that is inactivated by Ser21 phosphorylation, was dephosphorylated after HS. The GSK3α inhibitor CHIR99021 was able to abolish the effects of HS on sperm and their mitochondria. Taken together, our results demonstrate that HS affects sperm motility through downregulation of mitochondrial activity and ATP synthesis yield, which involves dephosphorylation of GSK3α and interference of mitochondrial remodeling.

## Introduction

The adverse effects of heat stress (HS) on animal reproductive functions have been studied extensively for many decades (Hansen, 2009; Kim et al., 2013). Exposure of the testes to an acute or chronic increase of temperature during the spermatogenic cycle can reduce the sperm number in the ejaculate and affect sperm parameters, such as motility, morphology, and plasma membrane integrity (Perez-Crespo et al., 2008; Kim et al., 2012, 2013; Zhang et al., 2012; Rao et al., 2016; Sabes-Alsina et al., 2016; Wang et al., 2016). After leaving the testes, sperm cells are vulnerable to environmental changes. In fact, sperm are sensitive to HS both in epididymis and female reproductive tract (Durairajanayagam et al., 2015; Sabes-Alsina et al., 2016). Nevertheless, the molecular basis of HS-induced decrease of sperm motility has not been entirely elucidated.

Mammalian sperm movement relies on large amounts of adenosine triphosphate (ATP), which is utilized by axonemal dynein to drive sperm motility (Odet et al., 2013). Mitochondria are the main ATP producers in cells. Mitochondria produce ATP via oxidative phosphorylation (OXPHOS), which takes place within the electron transport chain (ETC) (Pfeiffer et al., 2001). We previously demonstrated that OXPHOS is crucial for boar sperm motility (Guo et al., 2017). More than 1,500 proteins are localized in and/or on mitochondria (Sickmann et al., 2003). Most of them are encoded by the nuclear genome, synthesized on cytosolic ribosomes and imported into the mitochondria via a process named mitochondrial remodeling (Chacinska et al., 2009; Becker et al., 2012). Hence, the mitochondrial activity is dependent on the processes of transcription and translation as well as posttranslational changes of these proteins (Zorov et al., 2014). Previous studies have shown that mitochondrial dysfunction and oxidative stress are associated with HS (Chou et al., 1989; Downs and Heckathorn, 1998; Zhao et al., 2006; Slimen et al., 2014). However, the response of sperm mitochondria to HS and the underlying mechanisms are not fully elucidated.

Glycogen synthase kinase 3 (GSK3) is a serine/threonine protein kinase that mediates a large number of cellular processes. It is encoded by two genes that generate two related proteins: GSK-3α and GSK-3β (Kaidanovich-Beilin et al., 2012). It can be activated by Tyr279 phosphorylation and inactivated by Ser21 phosphorylation (Kaidanovich-Beilin and Woodgett, 2011). A direct relationship between Ser21 phosphorylation state of GSK3α and sperm motility has been found in human (Somanath et al., 2004; Bhattacharjee et al., 2015; Zhu et al., 2016), bovine (Vijayaraghavan et al., 2000), and porcine spermatogenesis (Aparicio et al., 2007; Bragado et al., 2010). GSK3 mediates the inhibition of PPP1R2, an inhibitory subunit of protein phosphatase 1 (PP1) that is known to play a key role in repressing sperm motility (Dacheux and Dacheux, 2014; Koch et al., 2015). Serine phosphorylation of GSK-3 increases significantly in spermatozoa during their passage through the epididymis where sperm changes from immotile to motile (Somanath et al., 2004; Koch et al., 2015). A significant increase in Ser21 phosphorylation of GSK3α was observed in boar sperm when temperature rose from 17 to 38°C (Aparicio et al., 2007). Recently, several studies indicated that GSK3 plays a pivotal role in the regulation of mitochondrial activity. In *Xenopus* oocytes, the activation of GSK3 was associated with the inhibition of mitochondrial ETC remodeling (Sieber et al., 2016). Conversely, an acute inhibition of GSK3 activated mitochondrial remodeling in the heart of mice (Nguyen et al., 2012). Nevertheless, whether and how GSK3α regulates sperm motility via inhibition of mitochondrial activity is not known.

Therefore, we hypothesized that the deleterious effects of high temperature in the female reproductive tract on sperm may be mediated by activation of GSK3α and interference of mitochondrial remodeling. To test this hypothesis, we use mature boar sperm as model to gain insight into the impacts of HS on mitochondrial function and sperm motility. High temperature can be achieved in the uterus of domestic species when exposed to continuously high environmental temperature. We compared the sperm under normal (37°C) and hyperthermia (42°C) conditions. Moreover, the dephosphorylation of GSK3α, mitochondrial function and mitochondrial remodeling were determined to reveal possible mechanisms. The results may provide new insights in understanding the mechanisms underlying HS-induced sperm damage.

## Materials and methods

### Ethics statement

The Institutional Animal Care and Use Committee (IACUC) of Nanjing Agricultural University approved all animal procedures. The “Guidelines on Ethical Treatment of Experimental Animals” (2006) No. 398 set by the Ministry of Science and Technology, China and the Regulation regarding the Management and Treatment of Experimental Animals” (2008) No. 45 set by the Jiangsu Provincial People's Government, was strictly followed during the slaughter and sampling procedures.

### Reagents

The JC-1 (5,5′,6,6′-tetrachloro-1,1′,3,3′tetraethylbenzymidazolyl carbocyanine iodide) mitochondrial membrane potential (ΔΨm) kits were purchased from Nanjing KeyGen Development Co., Ltd. (China). The MitoSOX Red (M36008), a specific fluorescent probe for superoxide produced by mitochondria was purchased from Invitrogen Co., Ltd., (Carlsbad, CA, USA) (Thermo Fisher Scientific, Hudson, NH, USA). The ATP determination kits (S0026) and the cell mitochondria isolation kits (C3601) were purchased from Beyotime Biotechnology (China). The GENMED kit (GMS50010) used for mitochondrial respiratory chain complex I activities measuring and the GENMED kit (GMS50007) used for mitochondrial respiratory chain complex IV activities determine, which were purchased from Genmed Scientifics Inc. (China). The ATP (A6419) was purchased from Sigma Chemical Co. (St. Louis, MO, USA). The GSK3α inhibitor (CHIR99021, S1263) was purchased from Selleck Chemicals LLC (Houston, TX, USA).

### Collection and culture of sperm

Twelve mature and healthy Duroc boars, aged 15–28 months were included in the study. The boars were housed in individual pens with straw bedding and received a standard balanced diet. Full ejaculates without the gel fraction were collected by the gloved-hand technique at a local artificial insemination center. The sperm-rich fraction was collected weekly from each boar using the gloved-hand technique. Following collection, the filtered semen of each ejaculate was extended with Beltsville Thawing Solution (BTS, Truadeco, Netherlands) at a ratio of 1:3. Each semen sample was treated separately. The semen was then incubated at 37 or 42°C under 5% CO_2_ atmosphere. To investigate whether ATP could improve the sperm motility, after 5.5 h 42°C treatment, 5 μM ATP was added into the culture medium for 0.5 h. To investigate the function of GSK3α, the sperm were challenged with or without 5 μM GSK3α inhibitor CHIR99021 for 6 h.

### Sperm motility analysis

Sperm motility was evaluated by a computer-assisted sperm motility analyzer (Sperm Vision 3.5, Minitube, Germany). Sperm motility was assayed by flow cytometry as previously described (Rodriguez et al., 2011). Briefly, samples were prepared and inserted into testing capillaries according to the SQA-Vb user's guide, and then placed into the SQA-chamber for 40 s. The total motility (%) and progressive motility (%) were measured.

### Measurement of sperm mitochondrial membrane potential (ΔΨm)

Sperm ΔΨm was measured using a JC-1 fluorescent probe. Sperm at 10 × 10^6^ cells/ml was incubated at 37° or 42°C for 6 h before analysis by flow cytometry according to previous publications (Lee et al., 2008; Marques et al., 2014). Briefly, treated sperm were centrifuged at 1,000 g for 5 min and then stained with 2.5 μg/ml JC-1 for 15 min at 37°C. Then, washed with ice-cold PBS two times, samples were analyzed by flow cytometry, and 10,000 events were acquired on flow cytometer. JC1 emissions from excitation at the 488 nm were collected at 525 nm (Fl1; JC1 green) and 585 nm (Fl2, JC1 orange). Gates, including the final gate for dye excluding cells were subjectively set based on the flow cytometry images, however, within each experiment the same gate settings were used to determine dye exclusion cohort percentiles changes that resulted from experimental maneuvers. Tests demonstrated that increasing or decreasing the stringency of the dye excluding gate selection which proportionally changed the % of cells within the gate, had very minimal effect on the percentile change induced by any given experimental manipulation within each experiment.

### Transmission electron microscopy (TEM) observation of mitochondria

Sperm was incubated at 37 or 42°C for 6 h and fixed with 2% glutaraldehyde, post-fixed with 1% osmium tetroxide, and embedded in resin. Ultrathin sections were cut and stained with uranyl acetate and lead citrate. Sperm ultrastructure was determined with a transmission electron microscope (Hitachi H-7650, Hitachi Technologies, Tokyo, Japan).

### Measurement of sperm mitochondrial ROS (mROS) contents

The intracellular generation of mitochondrial ROS was determined using MitoSOX red according to previous publications (Lee et al., 2008; Marques et al., 2014). This reagent penetrates live cells and selectively localizes to the mitochondria. Once in this location, the probe is oxidized by superoxide anion, generating a red fluorescence. For the assay, stock solutions of MitoSOX red was diluted in PBS and added to sperm at 10 × 10^6^ cells/ml to give final concentrations of 2 μM, and incubated for 15 min at 37°C with their treatments. Samples were then centrifuged for 5 min at 600 g, and the pellets were resuspended in 1 mL PBS and subsequently transferred to 5-mL FACS tubes. The MitoSOX red fluorescence was then measured on a flow cytometer. Argon laser excitation at 488 nm was coupled with emission measurements using 585/42 bandpass filters. Non-sperm-specific events were gated out and 10,000 cells were examined per independent sample.

### Measurement of sperm ATP levels

Sperm were collected by centrifugation and lysed by the addition of 300 μL of extraction buffer. The suspensions were centrifuged at 12,000 g for 5 min, and supernatants were maintained on ice until measurements were taken. ATP concentrations in 20 μL samples were determined using the ATP determination kit according to the manufacturer's instructions. Samples were measured in a Glomax 96 microplate luminometer (Promega Corporation, Madison, WI, USA). Serial dilutions were made from an ATP stock provided by the manufacturer for use as ATP standards. Each standard concentration was analyzed in triplicate for standard curve construction. The linearity of the relationship between bioluminescence and ATP concentration was initially tested over the range of 0.01–10 μM (*R*^2^ = 0.98).

### Determination of mtDNA copy number

Total genomic DNA was isolated and the mtDNA copy number was determined using real-time PCR as previously described (Guo et al., 2017). Primers specific for mtDNA (Accession No. NC_000845.1) were used for quantification (F: 5′-TCCTACTGGCCGTAGCATTCCT-3′, R: 5′-TTGAGGATGTGGCTGGTCGTAG-3′). Peptidylprolyl isomerase A (*PPIA*, also known as cyclophilin A) (Accession No. NM_214353.1) was chosen as a reference gene (F: 5′-GACTGAGTGGTTGGATGG-3′, R: 5′-TGATCTTCTTGCTGGTCTT-3′). All primers were synthesized by Generay Biotech Co., Ltd. (Shanghai, China). Real-time quantitative PCR was performed with a Mx3000P real-time PCR detection system (Stratagene, CA, USA). The amplification specificity of each gene was checked by melting curve analysis. Relative mtDNA copy number was calculated with 2^−ΔΔCt^ method.

### Mitochondrial respiratory chain complexes activity analysis

Mitochondrial respiratory chain complex I and IV activities were determined using GENMED kits by colorimetric method. Sperm protein was extracted and corrected to the same concentration. Mitochondrial respiratory chain complex I and IV activities were evaluated by GENMED kit according to the manufacturer's instruction. Briefly, the sperm were resuspended with lysed buffer, frozen at −70°C and thawed at 37°C three times to extract the mitochondrial proteins. The protein concentration in the lysate was determined using the BCA Protein Assay Kit (Pierce, Rockford, IL, USA). The absorbance was determined on a Smartspec™ Plus spectrophotometer (Bio-Rad). The activity of complex I-linked NADH-ubiquinone reductase was determined by measuring the reduction of ubiquinone to ubiquinol, which leads to a decrease in absorbance of NADH at 340 nm. The activity was measured with or without rotenone, a specific inhibitor of NADH-ubiquinone reductase. The specific activity of complex I was calculated by subtracting the rotenone-nonsensitive activity from the total activity and is expressed as μM NADH mg/min. Complex IV activity was measured by following the oxidation of reduced cytochrome *c* at 550 nm and was expressed as the first-order rate constant per milligram of protein.

### Protein extraction and western blot analysis

Sperm samples or mitochondria samples were homogenized in RIPA buffer (50 mM Tris-HCl pH 7.4, 150 mM NaCl, 1% NP40, 0.25% Na-deoxycholate, 1 mM PMSF, 1 mM sodium orthovanadate with Roche EDTA-free complete mini protease inhibitor cocktail, no. 11836170001). The protein concentration was measured with the BCA Protein Assay Kit (Pierce, Rockford, IL, USA) according to a previous publication (He et al., 2016). Fourty micrograms of protein extract were used for electrophoresis on a 15 or 10% SDS-PAGE gel. The following primary antibodies were used: rabbit anti-COX1 antibody (1:500, Bioworld, bs1636, China), mouse anti-COX4 antibody (1:500, Bioworld, MB0102, China), rabbit anti-HSP70 (1:500, Bioworld, bs2741, China), rabbit anti-Phospho-GSK3α (Ser21) antibody (1:1,000, Cell Signaling Technology, 9316, MA, USA), and rabbit anti-GSK3α antibody (1:1,000, Cell Signaling Technology, 9338, MA, USA). Protein loading controls for each experiment using rabbit anti-α-tubulin (1:10,000, Bioworld, bs1699, China) or mouse anti-β-actin antibody (1:1,000, Bioworld, AP0060, China). All the operations were carried out according to the recommended protocols provided by the manufacturers.

### Mitochondria isolation

Mitochondria were isolated from sperm using the Mitochondria Isolation Kit, C3601 (Beyotime Biotechnology, Shanghai, China). Briefly, sperm were collected, washed with PBS, and then suspended in ice-cold isolation buffer for 15 min. After the cells were homogenized, the homogenate was centrifuged at 600 g for 10 min at 4°C, and the supernatant was then centrifuged at 11,000 g for 10 min at 4°C. The mitochondria were collected in the sediments. All sediments were observed using a microscope with no mid-piece and tail of sperm. Moreover, we performed western blot analysis for COX1 and COX4 proteins in mitochondria lysate and lysate of sperm without mitochondria. No COX1 proteins in was detected in lysates of sperm without mitochondria indicated that this kit for isolating mitochondria in sperm was usable (data not shown).

### Immunofluorescence staining for p-GSK3α

Boar sperm were fixed in 4% neutral paraformaldehyde for 30 min, treated with TBS containing 0.5% Triton X-100 at room temperature for 1 h, blocked with 10% FCS and then incubated with rabbit anti-Phospho-GSK3α (Ser21) antibody (Cell Signaling Technology, 9316, MA, USA) overnight at 4°C. The tetramethylrhodamine-labeled goat anti-rabbit IgG (1:1.000, KPL Inc., Gaithersburg, MD, USA) were used as the second antibody. The 40, 6-diamidino-2-phenylindole (DAPI, Sigma Aldrich, MO, USA) was used as a marker for cell nuclei. Negative control sections were incubated with normal serum instead of primary antibodies. Mounted slides were visualized using a fluorescence microscope (Leica, DMI6000 B, Germany).

### Statistical analysis

All data are presented as mean ± SEM and were analyzed using independent samples *t*-test and Two-way ANOVA followed by LSD *post-hoc* test with SPSS 16.0 for windows. The method of 2^−ΔΔCt^ was used to analyze the real time PCR data. The differences were considered statistically significant when *P* < 0.05.

## Results

### HS-induced decrease of sperm motility

**Figure 1:**
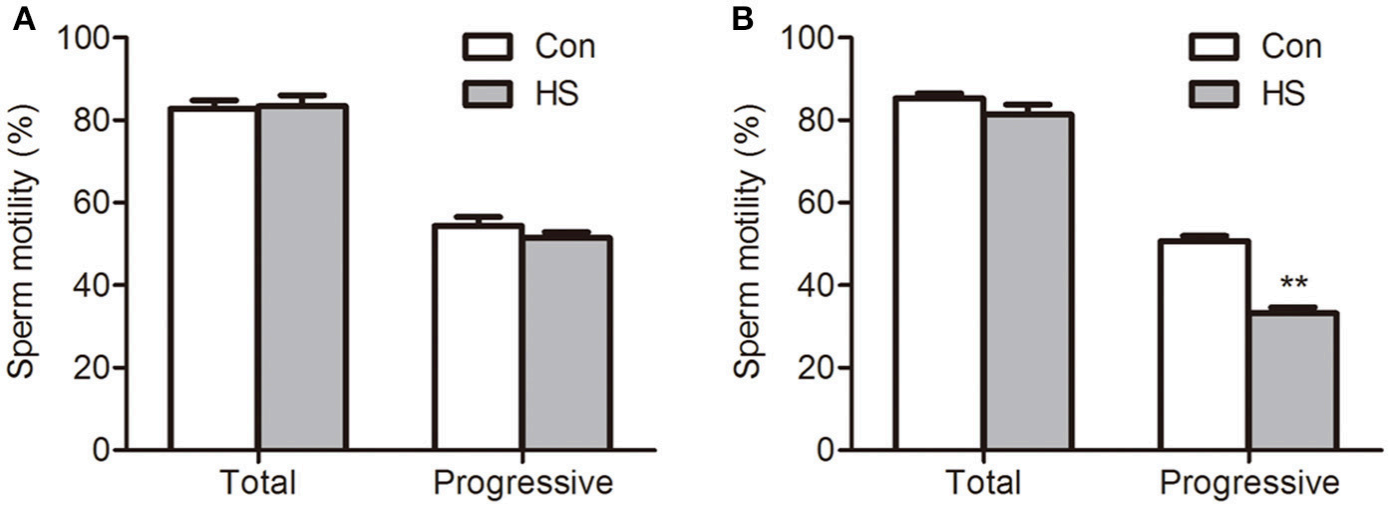
Effects of heat stress on sperm motility. Sperm were cultured at 37°C (Con) or 42°C (HS) for 3 h **(A)** and 6 h **(B)**. Values are expressed as the mean ± SEM, *n* = 6. Statistical analysis by two-tailed unpaired *t*-test. ^**^P < 0.01.

The boar sperm exposed to 42°C (HS) for 3 or 6 h did no significant effect in the percentage of total motility (Figure 1A). Progressive motility is the sperm's ability to move straight forward in a clearly defined direction. This ability is essential for spermatozoon movement in the female reproductive tract (Broekhuijse et al., 2012). In this study, exposed to 42°C (HS) for 3 h did no significant effect in the percentage of progressive motility sperm (Figure 1B). However, a significant (*t*-test, *P* < 0.01) decrease in the percentage of progressive motility sperm was detected after 6 h (Figure 1B).

### HS-induced decrease of mitochondrial biosynthesis

**Figure 2:**
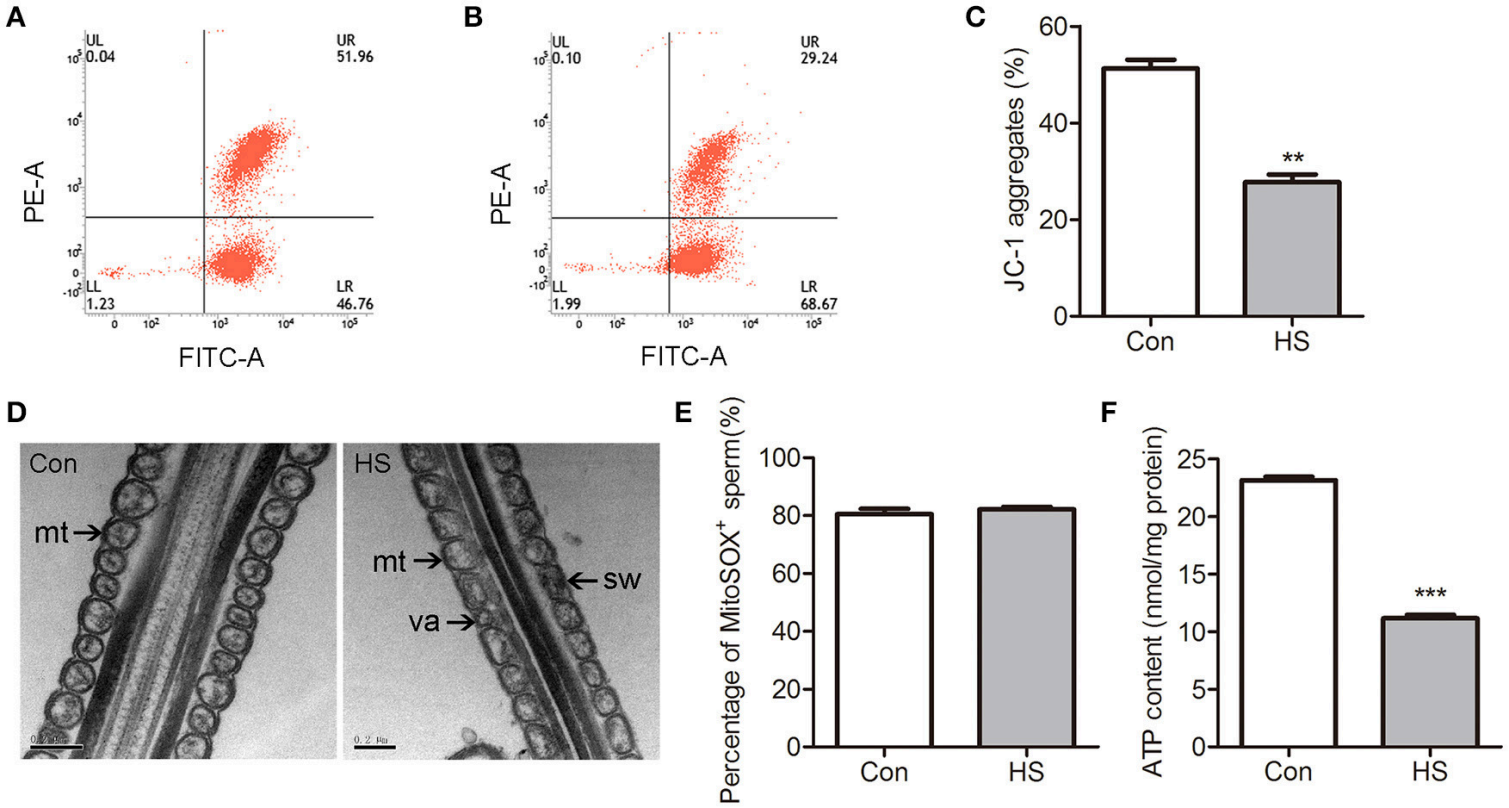
Effects of heat stress on mitochondrial ultrastructure and activity in sperm. Sperm were cultured at 37°C (Con) or 42°C (HS) for 6 h. (**A–C**) The mitochondria were staining with JC-1 and using flow cytometric analysis to evaluate the ΔΨm of Con **(A)** and HS **(B)** sperm. **(D)** The mitochondrial ultrastructure in Con and HS sperm were observed by electron microscopy. Swelling (sw) and vacuole (va) structures were recognized in HS-treated sperm mitochondria (mt). **(E)** The mROS contents in sperm using MitoSOX staining and flow cytometry analysis. **(F)** The ATP levels in sperm. mt: mitochondria. Values are expressed as the mean ± SEM, *n* = 6. Statistical analysis by two-tailed unpaired *t*-test. ^**^P < 0.01. ^***^P < 0.001.

Normal mitochondrial respiratory function is essential for high motility of sperm. The energy status of the mitochondria is depicted by the ΔΨm, which regulates the intact functional mitochondria and is directly associated with the motility of sperm (Marchetti et al., 2002). The mitochondrial probe JC-1 was used with flow cytometry to assess ΔΨm. The number of high ΔΨm was significant (*t*-test, *P* < 0.01) lower in HS than control group (Figures 2A–C).

Transmission electron microscopy was employed to evaluate mitochondrial ultrastructure in boar sperm. Most of the mitochondria contain clearly visible intact inner membrane, outer membrane and a well-defined inter membrane space are tightly packed into the midpiece from control (Figure 2D). In contrast, marked ultrastructural changes in the mitochondria including disorientation, swelling and vacuole structures were noted in HS-treated sperm (Figure 2D).

The mitochondria-specific superoxide fluorescent probe MitoSOX Red was applied to detect mROS production in sperm using flow cytometry. There was no significant difference in mROS levels between groups (Figure 2E). The level of ATP was significantly (*t*-test, *P* < 0.01) decreased in HS sperm (Figure 2F).

### HS-induced decrease of mitochondrial respiratory chain activity

**Figure 3:**
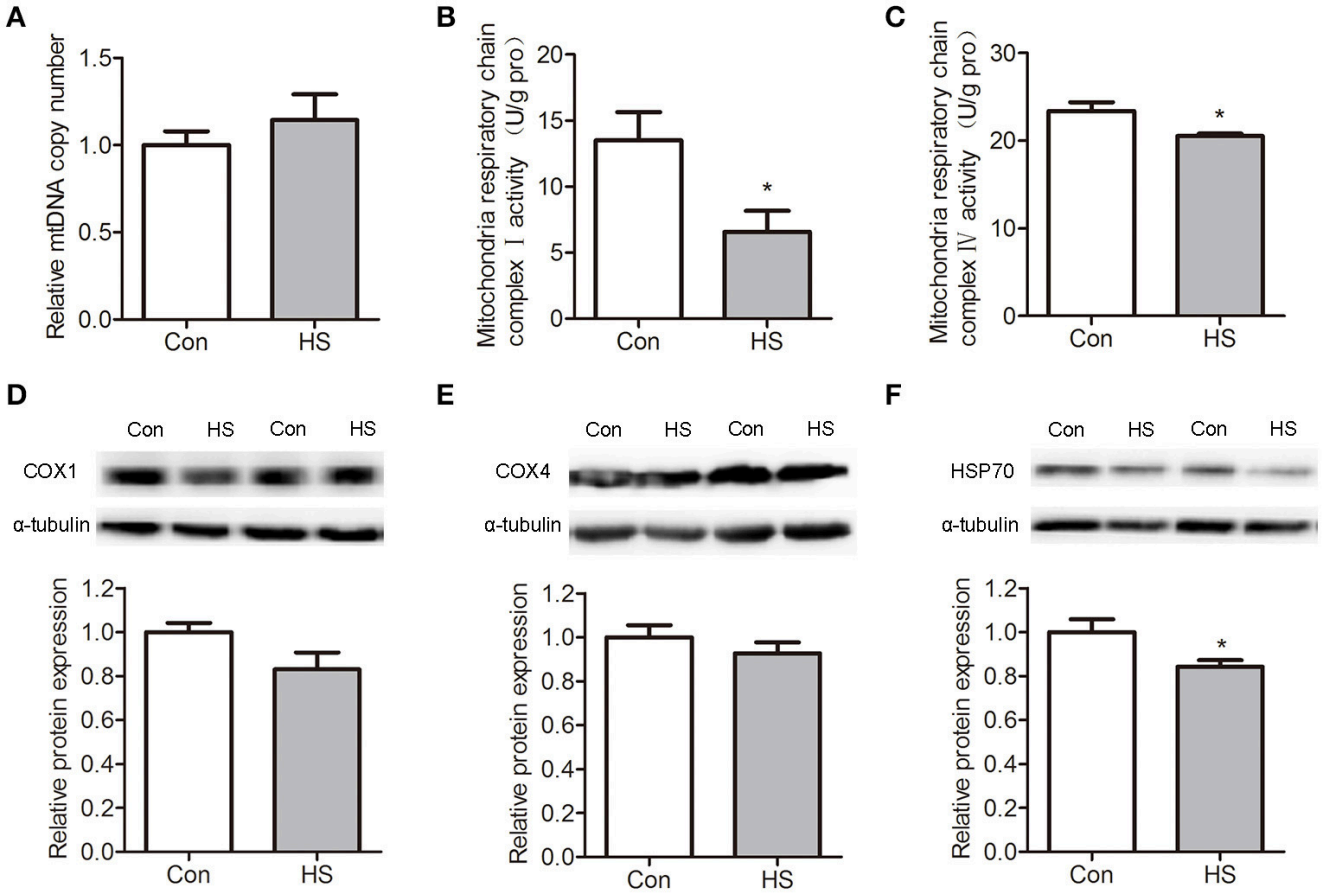
Effects of heat stress on mtDNA contents, respiratory chain activities and protein levels in sperm. Sperm were cultured at 37°C (Con) or 42°C (HS) for 6 h. **(A)** The relative mtDNA copy numbers were analyzed by real-time PCR. **(B–C)** The mitochondrial respiratory chain complex I activity **(B)** and mitochondrial respiratory chain complex IV activity **(C)** in Con and HS sperm. **(D–F)**. The levels of COX4 **(D)**, COX1 **(E)**, and HSP70 **(F)** protein in sperm were detected by western blot. Values are expressed as the mean ± SEM, *n* = 6. Statistical analysis by two-tailed unpaired *t*-test. ^*^P < 0.05.

The mtDNA content was measured by using quantitative real-time PCR assay. No significant difference was detected between control and HS groups (Figure 3A). Mitochondrial OXPHOS is carried out in the inner mitochondrial membrane by five enzymatic complexes of the ETC. We found that the mitochondrial respiratory chain complex I (NADH dehydrogenase, *t*-test, *P* < 0.05) and mitochondrial respiratory chain complex IV (cytochrome c oxidase, *t*-test, *P* < 0.05) activities were significantly lower in HS sperm than in control (Figures 3B–C).

The cytochrome c oxidase subunit 1 (COX1) is a protein encoded by mtDNA that participates in mitochondrial ETC and OXPHOS. Western blot results indicate that COX1 protein levels were no significant differences between groups (Figure 3D). The cytochrome c oxidase subunit 4 (COX4), which was synthesized in cytoplasm and translocated into mitochondria, showed no significant difference between groups (Figure 3E). The protein levels of heat shock protein 70 (HSP70) was significantly (*t*-test, *P* < 0.05) downregulated in HS sperm (Figure 3F).

### HS-induced decrease of mitochondrial protein transport

**Figure 4:**
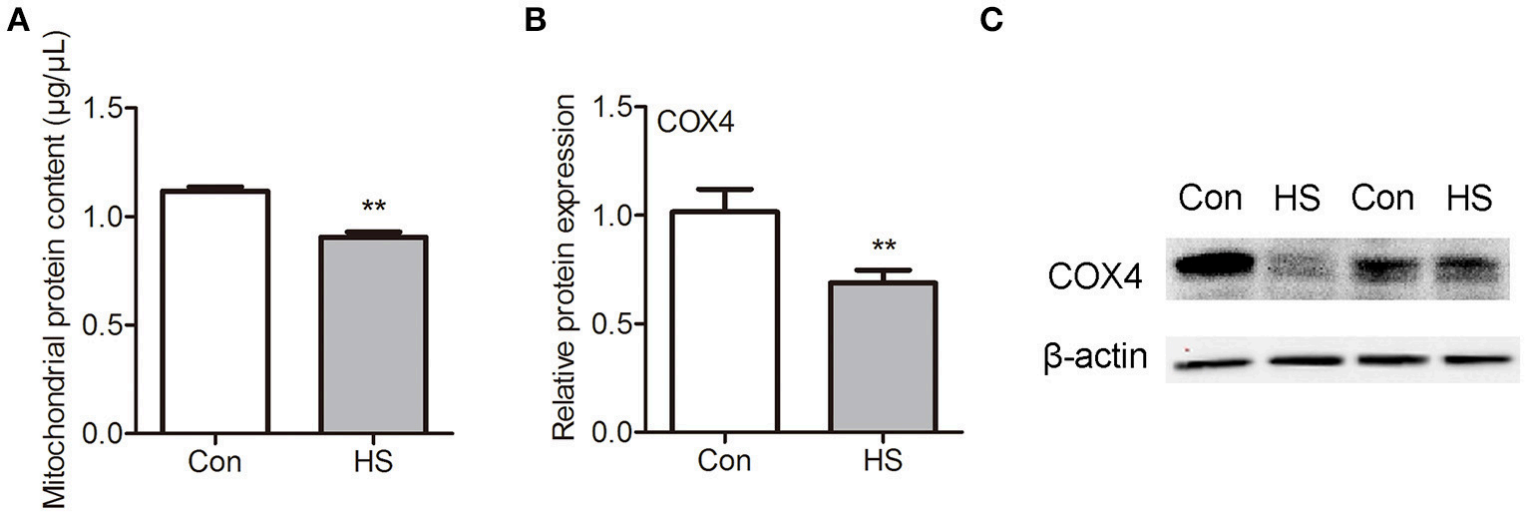
Effects of heat stress on mitochondria protein contents in sperm. Sperm were cultured at 37°C (Con) or 42°C (HS) for 6 h. **(A)** The mitochondrial protein contents in sperm. **(B,C)** The levels of COX4 protein in mitochondria of sperm. Values are expressed as the mean ± SEM, *n* = 4. Statistical analysis by two-tailed unpaired *t*-test. ^**^P < 0.01.

As most mitochondrial proteins are synthesized in cytoplasm and imported into mitochondria, we isolated sperm mitochondria by differential centrifugation and extracted the mitochondrial protein for further investigation. The mitochondrial protein contents were significantly (*t*-test, *P* < 0.05) lower in HS sperm (Figure 4A). Moreover, we performed western blot analysis for COX1 and COX4 proteins in mitochondria lysate and lysate of sperm without mitochondria. No COX1 proteins in was detected in lysates of sperm without mitochondria indicated that this kit for isolating mitochondria in sperm was usable (data not shown). However, we found the level of mitochondrial COX4 was significantly (*t*-test, *P* < 0.01) lower in HS group than in control (Figures 4B–C). These data confirmed that HS affects sperm mitochondria function through interference of mitochondrial protein transport and mitochondrial remodeling.

### HS-induced dephosphorylation of *p*-GSK3α (Ser21)

**Figure 5:**
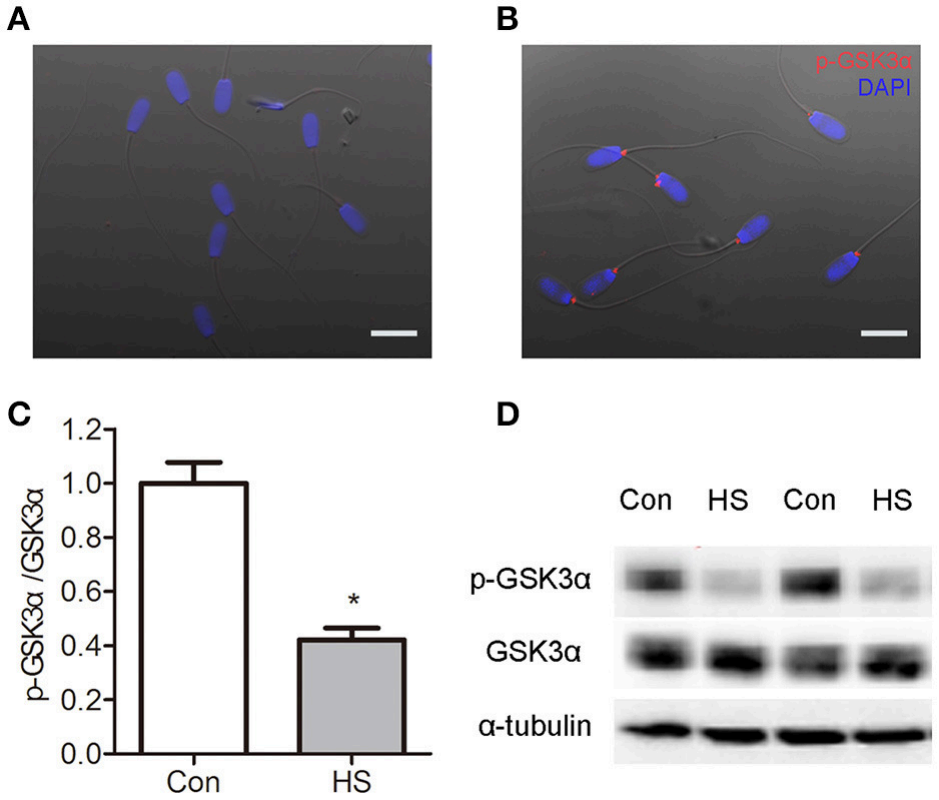
Effects of heat stress on dephosphorylation of GSK3α. Sperm were cultured at 37°C (Con) or 42°C (HS) for 6 h. **(A–B)** The distribution of p-GSK3α was detected by immunofluorescence staining in sperm. Boar sperm cultured without primary antibody **(A)** and with p-GSK3α **(B)**. Co-staining of the nucleus with DAPI is shown in blue. Scale bar: 10 μm. **(C,D)** The protein levels of p-GSK3α and total GSK3α were detected by western blot analyses. Values are expressed as the mean ± SEM, *n* = 6. Statistical analysis by two-tailed unpaired *t*-test. ^*^P < 0.05.

The GSK3α is a negative regulator of sperm motility and can be inactivated by Ser21 phosphorylation. So we examined the localization of p-GSK3α in boar sperm by immunofluorescence staining. Intense staining was observed in the posterior portion of the head, and joint of flagellum (Figures 5A–B). The levels of p-GSK3α and total GSK3α were detected. The results showed that levels of p-GSK3α were lower in HS sperm compared with control (Figures 5C–D). The ratio of p-GSK3α to total GSK3α was significantly (*t*-test, *P* < 0.05) decreased in HS sperm (Figure 5C). Moreover, the levels of p-GSK3β and total GSK3β were detected. There was no significant difference in levels of p-GSK3β, total GSK3β as well as rate of p-GSK3α/GSK3α between groups (data not shown).

### GSK3α participates in HS-induced reduction of sperm motility and mitochondrial activity

**Figure 6:**
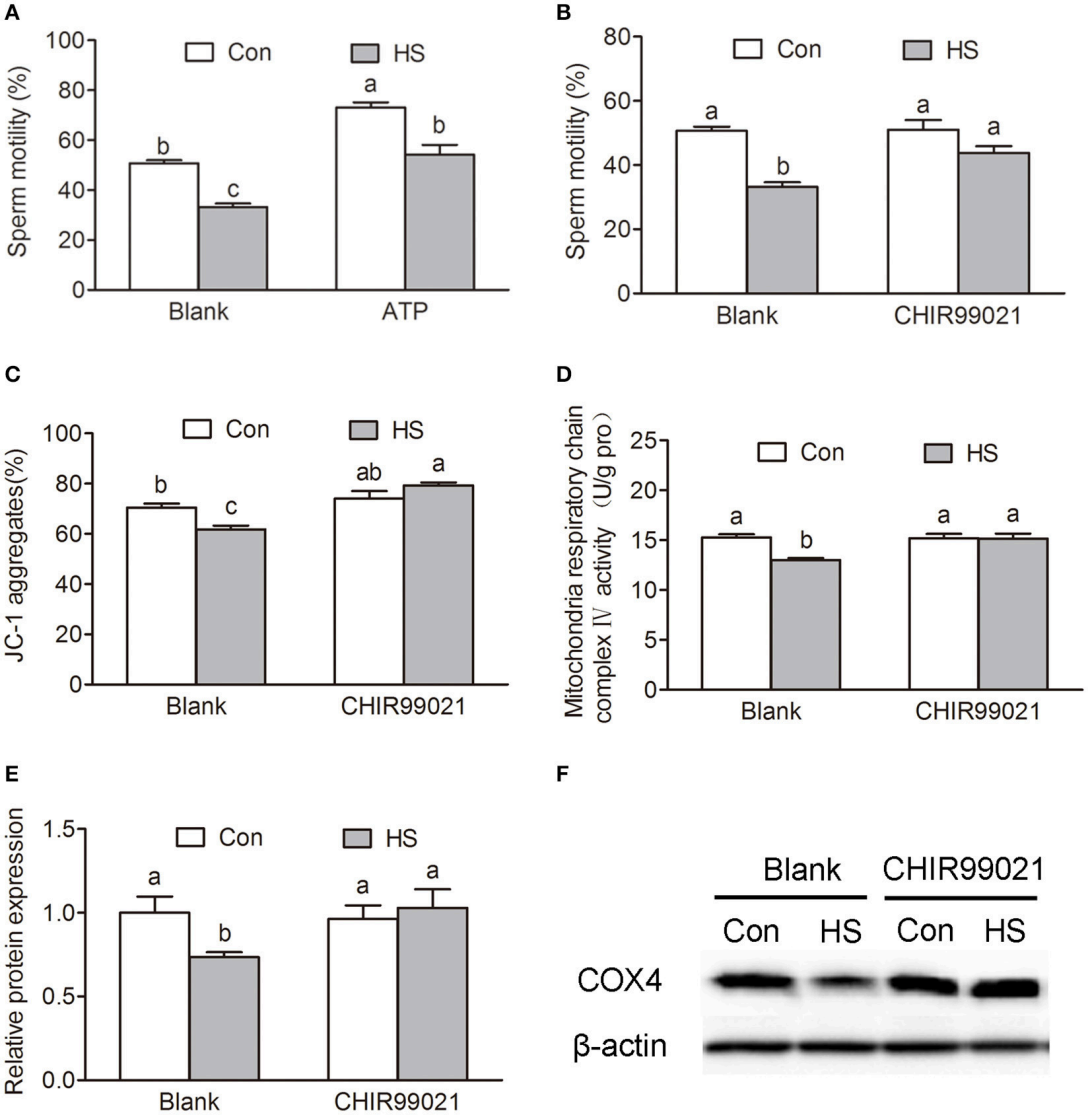
Roles of GSK3α in heat stress-induced reduction of sperm motility. **(A)** Sperm were cultured at 37°C (Con) or 42°C (HS) for 6 h and then add 10 mM ATP or saline for 30 min. The sperm motility was measured by sperm quality analyzer. **(B–D)** Sperm were cultured at 37 or 42°C with or without 100 nM CHIR99021 for 6 h. Sperm motility **(B)**, mitochondrial membrane potential **(C)** and mitochondrial respiratory chain complex IV activity **(D)** were determined. **(E,F)** The levels of mitochondrial COX4 protein in sperm were detected by western blot. Values are expressed as the mean ± SEM, *n* = 6. Statistical analysis by Two-way ANOVA followed by LSD *post-hoc* test. Values with different superscripts are significantly different from each other (*P* < 0.05).

The ATP synthesis in mitochondria via OXPHOS is essential for sperm motility. Sperm in HS group showed a significant decrease in progressive motility (Two-way ANOVA, *P* < 0.01, Figure 1A) and ATP content (Two-way ANOVA, *P* < 0.01, Figure 2F). Remarkably, the decrease of progressive motility was blocked by supplement of ATP in culture media for 30 min (Two-way ANOVA, *P* < 0.05, Figure 6A). Moreover, the decrease of progressive motility induced by HS can be blocked by GSK3α inhibitor, CHIR99021 (Figure 6B). Furthermore, CHIR99021 treatment significantly reversed HS-induced decrease of ΔΨm (Two-way ANOVA, *P* < 0.05, Figure 6C) and mitochondrial respiratory chain complex IV activity (Two-way ANOVA, *P* < 0.05, Figure 6D). The inhibition of COX4 transport into mitochondria by HS, was also significantly (Two-way ANOVA, *P* < 0.05) reversed by CHIR99021 (Figures 6E–F). These data confirmed that HS affects sperm progressive motility and mitochondria function through activation of GSK3α.

## Discussion

**Figure 7:**
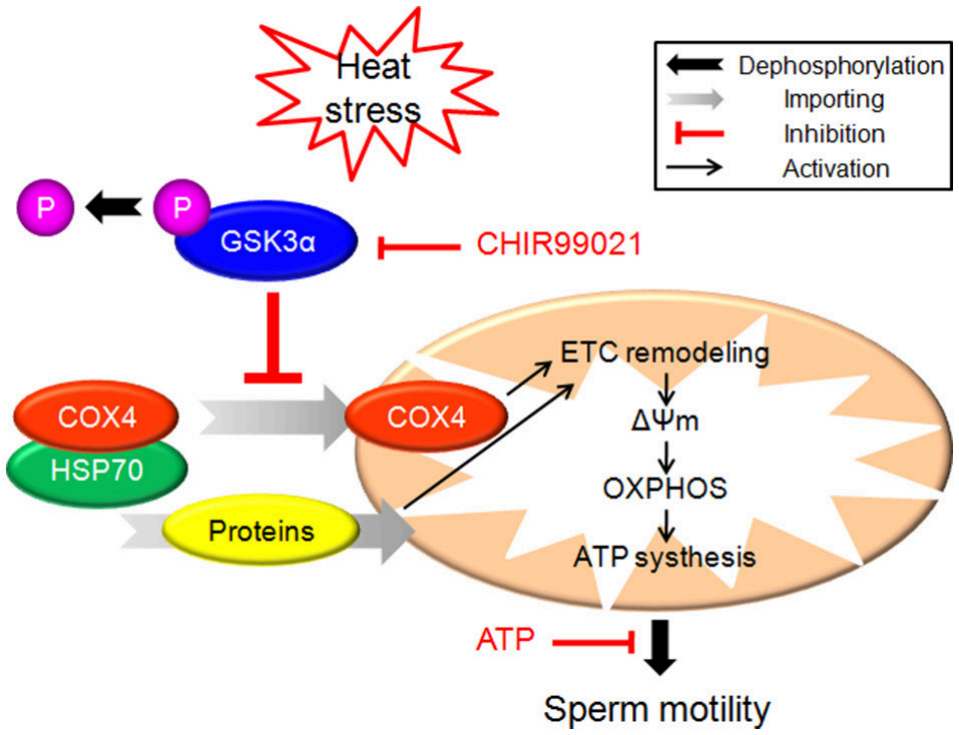
The schematic diagram demonstrates intracellular signaling events in HS-induced mitochondrial dysfunction and reduction of sperm motility in sperm.

In the present study, exposure of boar sperm to hyperthermic conditions (42°C) for 6 h induced decrease of progressive motility. HS induced sperm mitochondrial dysfunction which is indicated by marked ultrastructural changes, decrease of ΔΨm, mitochondrial respiratory chain complex I and IV activities as well as ATP synthesis yield. The dephosphorylation of GSK3α and impaired mitochondrial transport of proteins may be involved in the mechanisms underlying HS action. Our work suggests a novel role of GSK3α in the regulation of sperm motility. The schematic signaling pathway suggested by our data is presented in Figure 7.

Previous studies have noticed the impacts of HS on spermatogesis in testes (Perez-Crespo et al., 2008; Kim et al., 2012; Zhang et al., 2012; Durairajanayagam et al., 2015), yet the effect of HS on mature sperm are less concerned. After leaving the testes, sperm cells are vulnerable to environmental changes. Transient scrotal hyperthermia affects sperm DNA integrity, sperm apoptosis, and sperm protein expression (Kim et al., 2013; Rao et al., 2016). High temperature can be achieved in the female reproductive tract such as uterus (De Rensis and Scaramuzzi, 2003). The maximum temperature recorded in the reproductive tract of an hyperthermic female pig before fertilization was 40.7°C (Ingram and Legge, 1970). Rabbit spermatozoa parameters, including metabolic activity and motility, are largely modified by a short exposure to hyperthermic conditions (Sabes-Alsina et al., 2016). In the present study, HS induced decrease of sperm motility and ATP content in the sperm. Exogenous ATP can markedly restore the motility of hamster sperm pretreated with mitochondrial OXPHOS blockers (Bhattacharyya and Pakrashi, 1993). In the present study, exogenous ATP abolished these alterations, which suggests that reduction in ATP synthesis yield is the committed step in HS-induced decrease of sperm motility.

Mammalian sperm movement relies on large amount of ATP, which is utilized by axonemal dynein to drive sperm motility (Odet et al., 2013). OXPHOS is carried out in the inner mitochondrial membrane by the five enzymatic complexes of the mitochondrial ETC (Pfeiffer et al., 2001). Studies showed that mitochondrial activity is sensitive to high temperature (Chou et al., 1989). Mitochondrial disorders are clinical syndromes associated with abnormality of OXPHOS system (Becker et al., 2012). Previous studies have shown that mitochondrial dysfunction and oxidative stress are associated with HS (Downs and Heckathorn, 1998; Zhao et al., 2006). Here, we found that the ΔΨm, mitochondrial respiratory chain complex I and IV activities were significantly lower in HS sperm, which agrees with our previous report that OXPHOS activity is lower in less motile boar sperm (Guo et al., 2017). These results provide evidence that HS affects sperm motility through induction of mitochondrial dysfunction.

Most mitochondrial proteins are synthesized on cytosolic ribosomes and imported into mitochondria (Chacinska et al., 2009; Becker et al., 2012). Recently, several studies indicate that GSK3 plays a pivotal role in mitochondrial activity. In the present study, p-GSK3α was observed in the posterior portion of the sperm head, and the joint of flagellum. Moreover, the p-GSK3α levels were lower in HS group compared with control group. Previous *in vitro* studies have suggested that GSK3 binds to outer mitochondrial membrane proteins and regulates mitochondrial membrane potential and permeability (Tanno et al., 2014). GSK3 inhibition with indirubin-3′-oxime decreased mitochondrial permeability transition pore (Masgras et al., 2012). Nguyen et al. demonstrated that increase of 16 proteins in the mitochondrial fraction and mitochondrial remodeling after acute inhibition of GSK in heart of mice (Nguyen et al., 2012). Decrease of insulin signaling initiates ETC remodeling and mitochondrial respiratory quiescence through activation of GSK3 in *Xenopus* oocytes (Sieber et al., 2016). In the present study, we found that mitochondria protein contents were significantly decreased after HS accompanied with decrease of p-GSK3α. Notably, COX4, a component of cytochrome c oxidase that is synthesized in cytoplasm and translocated into mitochondria, was significantly lower in HS group than in control. To gain further insight into this process, we used a GSK3α inhibitor to check whether HS-induced interference of mitochondrial remodeling is achieved through dephosphorylation and activation of GSK3α. Treatment with GSK3α inhibitor blocked the inhibition of HS-induced mitochondrial remodeling. Further, the GSK3α inhibitor was able to abolish all the effects of HS on sperm including decrease of mitochondrial membrane potential, mitochondrial respiratory chain complex and mitochondrial respiratory chain complex IV activities, as well as a decrease of ATP levels. Taken together, these data confirm that HS affects sperm mitochondria function through activation of GSK3α and inhibition of mitochondrial proteins transport.

Studies have shown that HSPs affect mitochondrial function in multiple ways. HSPs act as molecular chaperones in the import of cytosolic proteins into mitochondria (Terada et al., 1995; Baker et al., 2007). The formation of the mature cytochrome c oxidase (complex IV) involves the association of nuclear- and mitochondria-encoded subunits. A complex of COX4 and mitochondrial HSP70 plays an important role in the assembly of the cytochrome c oxidase (Bottinger et al., 2013). In the present study, the levels of HSP70 in sperm were downregulated after HS. The downregulation of HSP70 may participate in HS-induced inhibition of proteins mitochondrial translocation.

Taken together, HS induced activation of GSK3α, which inhibits mitochondrial proteins transport and mitochondrial function in boar sperm. As a result, the synthesis of ATP was decreased which leads to a decrease of sperm progressive motility. Notably, a GSK3α inhibitor was able to abolish all the effects of HS on sperm. These data confirm that HS affects sperm motility through modulating mitochondrial activity and ATP synthesis yield. The dephosphorylation of GSK3α and mitochondrial remodeling may be involved in the mechanisms. The results will provide new insights in understanding the mechanisms underlying the sperm damage induced by high temperature in the female reproductive tract.

## Author contributions

YG carried out the experiments, participated in the data collection and interpretation, and drafted the manuscript. HG, ZZ, and HZ prepared materials and participated in the data collection. RZ participated in design, manuscript editing and revised the manuscript. BH contributed conception, experimental design, data interpretation, and finalized the manuscript.

### Conflict of interest statement

The authors declare that the research was conducted in the absence of any commercial or financial relationships that could be construed as a potential conflict of interest.
