# Supplementary figures and images for: Heat Stress Reduces Sperm Motility via Activation of Glycogen Synthase Kinase-3α and Inhibition of Mitochondrial Protein Import
Source: Front Physiol. 2017 Sep 22;8:718. doi: 10.3389/fphys.2017.00718 (PMC5615227; doi:10.3389/fphys.2017.00718)

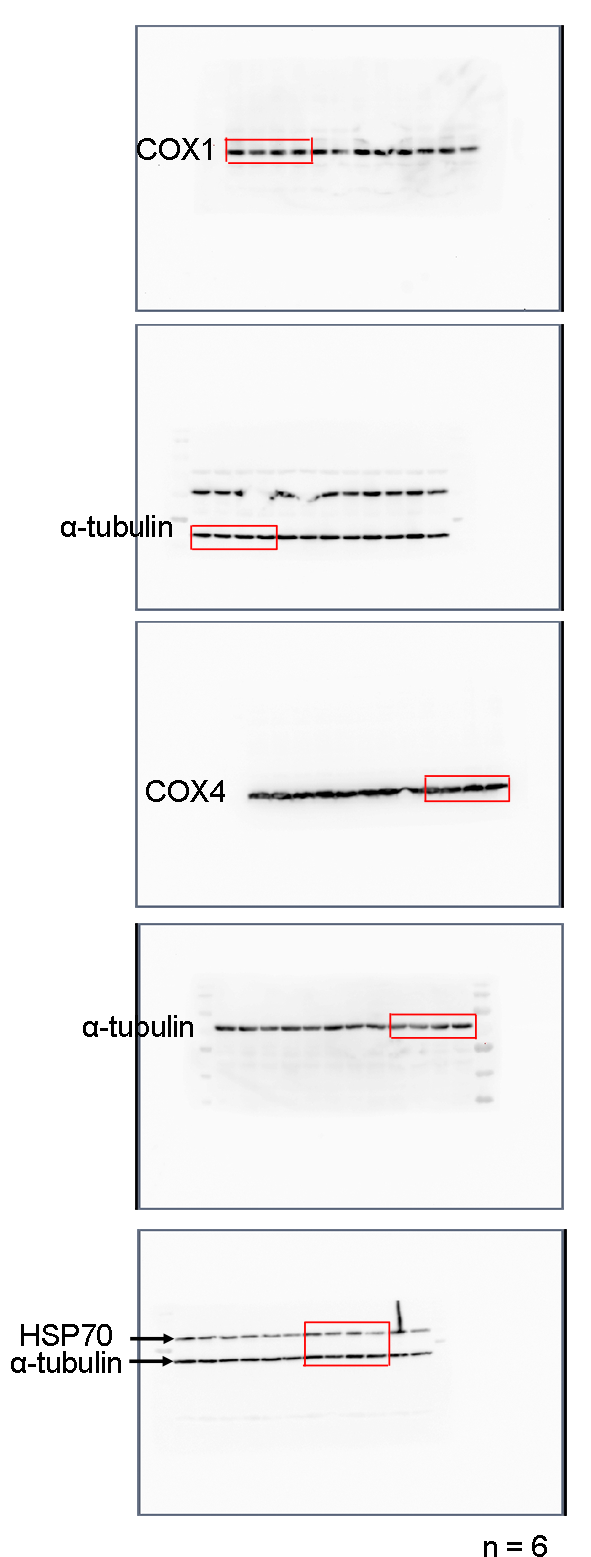

Supplement: Supplementary file 1 [file Image1.TIF]

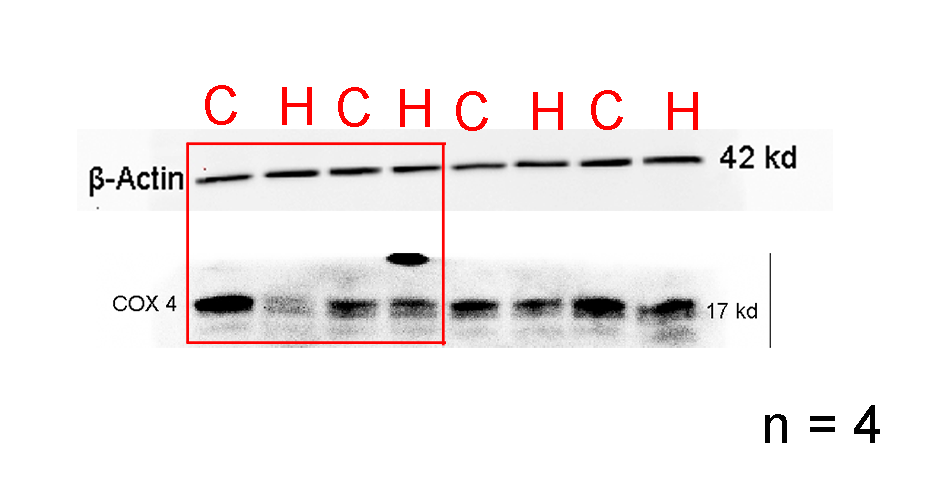

Supplement: Supplementary file 2 [file Image2.TIF]

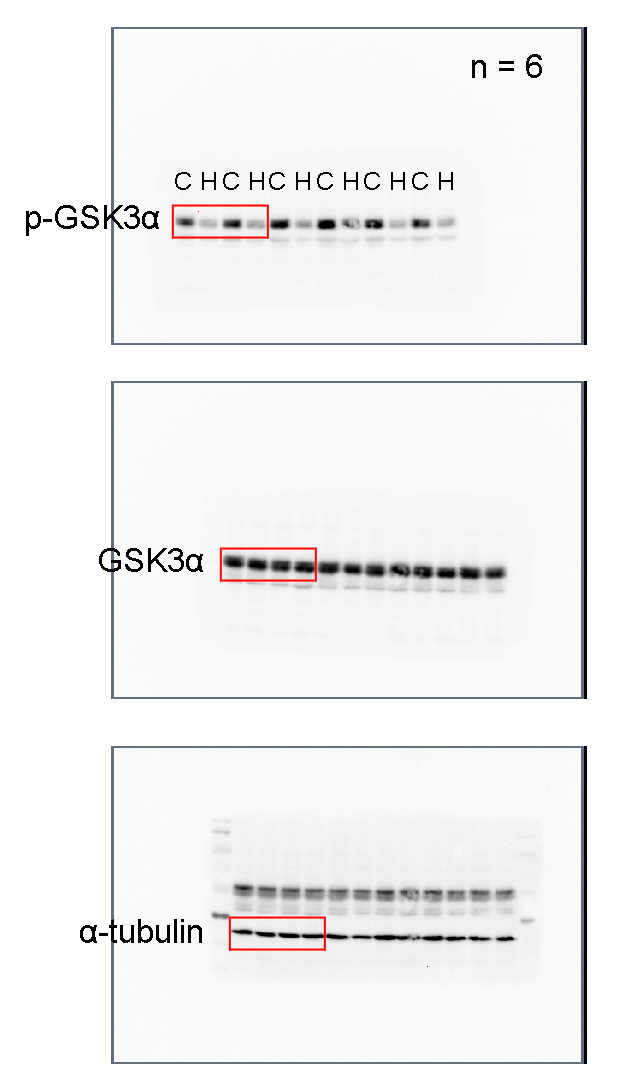

Supplement: Supplementary file 3 [file Image3.TIF]

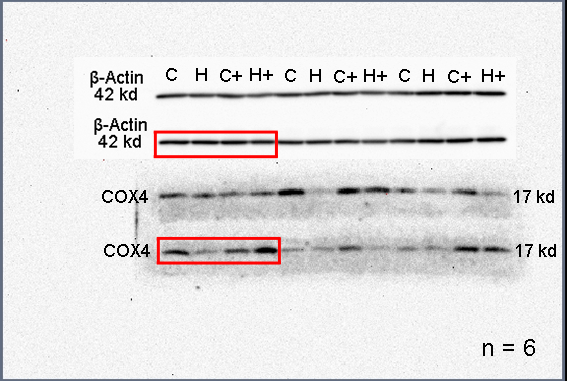

Supplement: Supplementary file 4 [file Image4.TIF]
