# Supplementary material for: Heat Stress Reduces Sperm Motility via Activation of Glycogen Synthase Kinase-3α and Inhibition of Mitochondrial Protein Import
Source: Front Physiol. 2017 Sep 22;8:718. doi: 10.3389/fphys.2017.00718 (PMC5615227; doi:10.3389/fphys.2017.00718)
Supplement: Supplementary file 5 [file DataSheet1.DOCX]

**Figure legends of supplementary images**

**Figure S1** The levels of COX1, COX4 and HSP70 proteins in sperm cultured at 37°C (C) or 42°C (H) for 6 h. n=6. Bands with red border around are shown in Figure 3.

**Figure S2** The levels of COX4 protein in mitochondria of sperm cultured at 37°C (C) or 42°C (H) for 6 h. n=4. Bands with red border around are shown in Figure 4.

**Figure S3** The protein levels of p-GSK3α and total GSK3α in sperm cultured at 37°C (C) or 42°C (H) for 6 h. n=6. Bands with red border around are shown in Figure 5.

**Figure S4** The levels of mitochondrial COX4 protein in sperm cultured at 37°C or 42°C with or without 100 nM CHIR99021 for 6 h. n=6. C: 37°C; H: 42°C; C+: 37°C with CHIR99021; H+: 42°C with CHIR99021. Bands with red border around are shown in Figure 6.
